# The Relationships between Workaholism and Symptoms of Psychiatric Disorders: A Large-Scale Cross-Sectional Study

**DOI:** 10.1371/journal.pone.0152978

**Published:** 2016-05-18

**Authors:** Cecilie Schou Andreassen, Mark D. Griffiths, Rajita Sinha, Jørn Hetland, Ståle Pallesen

**Affiliations:** 1 Department of Psychosocial Science, University of Bergen, Bergen, Norway; 2 Centre of Competence, Bergen Clinics Foundation, Bergen, Norway; 3 Psychology Division, Nottingham Trent University, Nottingham, United Kingdom; 4 Yale Stress Center, Yale University School of Medicine, New Haven, CT, United States of America; National Center of Neurology and Psychiatry, JAPAN

## Abstract

Despite the many number of studies examining workaholism, large-scale studies have been lacking. The present study utilized an open web-based cross-sectional survey assessing symptoms of psychiatric disorders and workaholism among 16,426 workers (M_age =_ 37.3 years, SD _=_ 11.4, range = 16–75 years). Participants were administered the Adult ADHD Self-Report Scale, the Obsession-Compulsive Inventory-Revised, the Hospital Anxiety and Depression Scale, and the Bergen Work Addiction Scale, along with additional questions examining demographic and work-related variables. Correlations between workaholism and all psychiatric disorder symptoms were positive and significant. Workaholism comprised the dependent variable in a three-step linear multiple hierarchical regression analysis. Basic demographics (age, gender, relationship status, and education) explained 1.2% of the variance in workaholism, whereas work demographics (work status, position, sector, and annual income) explained an additional 5.4% of the variance. Age (inversely) and managerial positions (positively) were of most importance. The psychiatric symptoms (ADHD, OCD, anxiety, and depression) explained 17.0% of the variance. ADHD and anxiety contributed considerably. The prevalence rate of workaholism status was 7.8% of the present sample. In an adjusted logistic regression analysis, all psychiatric symptoms were positively associated with being a workaholic. The independent variables explained between 6.1% and 14.4% in total of the variance in workaholism cases. Although most effect sizes were relatively small, the study’s findings expand our understanding of possible psychiatric predictors of workaholism, and particularly shed new insight into the reality of adult ADHD in work life. The study’s implications, strengths, and shortcomings are also discussed.

## Introduction

Workaholism has been defined as “being overly concerned about work, driven by an uncontrollable work motivation, and to investing so much time and effort to work that it impairs other important life areas” [1] (p. 8). Research into this timely topic has heavily expanded over the past few decades [2,3], and concerns have been raised regarding the downsides of workaholism [4,5]. In order to prevent workaholism developing, there is a need to identify factors involved with this compulsive work pattern–especially since modern technology (i.e., laptops, tablets, smartphones) has blurred the natural lines between home and the workplace.

Given this evolving context, the present study aimed to identify risk factors associated with workaholism, and to enrich the existing literature in several ways. Previous workaholism research has often used invalid measures, small samples, and insufficient theoretical frameworks [1,6,7]. In this study, a contemporary theoretical framework of addiction to conceptualize workaholism was applied, and validated scales were utilized to investigate whether several psychiatric symptoms were related to workaholism among a large sample of employees.

The latest edition of the *Diagnostic and Statistical Manual of Mental Disorders* (DSM-5) reconceptualized addictive behavior to include behavioral addictions akin to more traditional drug addictions [8]. Two profound changes were made: (i) Gambling Disorder (formerly pathological gambling) was reclassified as a behavioral addiction rather than a disorder of impulse control [9], (ii) and Internet Gaming Disorder was introduced into Section 3 of the DSM-5 (Emerging Measures and Models) [8]. However, at present, although these changes represent a substantial recognition of behavioral addictions in general, most potentially addictive behaviors are not yet formally defined as such–including workaholism.

As the line between excessive enthusiasm and a genuine addiction is difficult to define, scholars have typically used specific criteria to define the border between addictive and non-addictive behavior [10]. These criteria involve being totally preoccupied by work (salience), using work to alleviate emotional stress (mood modification), gradually working longer and longer hours to get the same mood modifying effects (tolerance), suffering emotional and physical distress if unable to work (withdrawal), sacrificing other obligations (personal relationships with partner and children, social activities, exercising, etc.) because of work (conflict), desiring or attempting to control the number of hours spent working without success (relapse), and suffering some kind of harm or negative consequence as either a direct or indirect result of the excessive working (problems) [11,12]. Because previous workaholism scales did not cover these addiction components, the seven-item Bergen Work Addiction Scale (BWAS) was specifically developed in order to assess this behavior using the same criteria as other addictions [13]. Consequently, the BWAS is based on and embedded within general addiction theory [10], and has demonstrated robust psychometric properties across studies in different countries [13–15].

Via mobile technology hardware, work is highly accessible to anyone and anywhere, and has the potential to facilitate and enhance workaholism tendencies [16,17]. However, there has been a perceivable paucity in the number of reliable prevalence estimates of workaholism. Systematic reviews and meta-analyses tentatively report estimates from 5% to over 25% [14,18]. According to a recent (and, to date, only) nationally representative study of Norwegian workers, 8.3% were categorized as workaholics [14]. Research has also shown that age is inversely related to workaholism [14,19]. Although a few studies have reported gender differences [20,21], workaholism appears to be unrelated to both gender and marital status [2,14,19]. Research has further demonstrated that higher education and having managerial duties are associated with workaholism [13,19,20,22,23]. A few studies have reported higher levels of workaholism in certain lines of work (e.g., commercial trade, agriculture, medicine, communication, consultancy, etc.) as well as sectors (private and self-employment) [19,20,22–24]. For some, workaholism has been described as a money disorder [25], and one study associated it with having a higher income [20]. This has good face validity because working hard usually means increased salary/earnings. Given these findings, it is expected that younger, well-educated workers, in self-employed and private sector, with managerial responsibilities and higher income will report higher scores on the Bergen Work Addiction Scale in the present study (Hypothesis 1).

Research have consistently demonstrated that Attention-Deficit/Hyperactivity Disorder (ADHD) increases the risk of various chemical and non-chemical addictions [26]. However, this psychiatric disorder has never been empirically examined (or theoretically associated with) workaholism. ADHD is prevalent in 2.5–5% of the adult population, and is typically manifested by inattentiveness and lack of focus, and/or impulsivity, and excessive physical activity [8,26]. Individuals with ADHD may often stop working due to their disorder, and may have trouble in getting work health insurance as they are regarded as a risk group [26]. For this reason, the present authors hypothesize that individuals with ADHD may compensate for this by over-working to meet the expectations required to hold down a job. Although this is a contentious issue, there are a number of reasons why ADHD may be relevant to workaholism.

Firstly, the present authors argue that the *inattentive nature* of individuals with ADHD causes them to spend time beyond the typical working day (i.e., evenings and weekends) to accomplish what is done by their fellow employees within normal working hours (i.e., the compensation hypothesis). In addition, as they may have a hard time concentrating while at work due to environmental noise and distractions (especially office work in open landscape environments), they might find it easier to work after co-workers have left their working environment or work from home. Their attentive shortcomings may also cause them to overly check for errors on the tasks given, since they often experience careless mistakes due to their inattentiveness [26]. This may cause a cycle of procrastination, work binges, exhaustion, and–in some cases–a fear of imperfection. Although ADHD is associated with lack of focus, such individuals often have the ability to hyper-focus once they find something interesting–often being unable to detach themselves from the task (e.g., flow) [27,28].

Secondly, the present authors’ argue that the *impulsive nature* of individuals with ADHD causes them to say ‘yes’ and taking on many tasks without them thinking ahead, and taking on more work than they can realistically handle–eventually leading to workaholic levels of activity. Thirdly, it is also argued that the *hyperactive nature* of individuals with ADHD and the need to be constantly active without being able to relax, causes such individuals to keep on working in an attempt to alleviate their restless thoughts and behaviors. Consequently, work stress might act as a stimulant, and they may choose active (and often multiple) jobs with high pressure, deadlines and activity (e.g., media, sales, restaurant work)–where they have the opportunity to multitask and constantly shift between tasks (e.g., Type-A personality behavior) [26,29]. In line with this, Type-A personality has often been associated–and sometimes used interchangeably–with workaholism in previous research [2,30]. This line of reasoning also relates to the workaholic type portrayed by Robinson [31], in which he actually denoted “attention-deficit workaholics” (who tend to start many projects but become bored easily and need to be stimulated at all times). His description of the “relentless” type also corresponds well with ADHD symptoms (i.e., unstoppable in working fast and meeting deadlines, often with many projects going on simultaneously). In other words, these types may utilize work pressure to obtain focus, constantly seeking stimulation, crisis, and excitement–and therefore like risky jobs.

Finally, people with ADHD are often mistaken as being lazy, irresponsible, or unintelligent because of their difficulties with planning, time management, organizing, and decision-making [26]. Feeling misunderstood might cause individuals with ADHD to push themselves to prove these misconceptions as wrong–and resulting in an excessive and/or compulsive working pattern. Such individuals are often intelligent, but may feel forced or motivated to start up their own business (i.e., entrepreneurs), as they find it troubling to adjust to standard work schedules or organizational boundaries. Previous research has highlighted that workaholism is prevalent among entrepreneurs and the self-employed [24,32]. Often failing in other aspects of life (e.g., family) [26], work for such individuals may become even more important to them (e.g., self-efficacy). In accord with the aforementioned line of reasoning and findings, it is hypothesized that ADHD symptoms will be positively associated with workaholism in the present study (Hypothesis 2).

Obsessive-Compulsive Disorder (OCD) is another underlying psychiatric disorder that increases the likelihood of developing an addiction [33]. Full-blown OCD occurs in approximately 2–3% of children and adults, and is commonly manifested by intrusive thoughts and repetitive behaviors of checking, obsessing, ordering, hoarding, washing, and/or neutralizing [8,34,35]. It has been suggested that addictive behaviors might represent a coping and/or escape mechanism of OCD symptoms, or as an OCD-behavior that eventually becomes an addiction in itself [36]. Previous workaholic typologies have incorporated the “compulsive-dependent” and “perfectionistic” types [37], and some empirical studies have demonstrated that obsessive-compulsive traits are present among workaholics [2,38,39]. The OCD tendency of having the need to arrange things in a certain way (i.e., a strong need for control) and obsessing over details to the point of paralysis–may predispose workers with such traits to develop workaholic working patterns [31,37,40,41]. Therefore, it is hypothesized that OCD symptoms will be positively related to workaholism in the present study (Hypothesis 3).

Other psychiatric disorders such as anxiety and depression may also increase the risk of developing an addiction [33]. Approximately 30% of people will suffer from an anxiety disorder in their lifetime, and 20% will have at least one episode of depression [34,35]. These conditions often occur simultaneously, as most people who are depressed also experience acute anxiety [36]. Anxiety and/or depression can lead to addiction, and vice versa [36]. A number of studies have previously reported a link between anxiety, depression, and workaholism [2,7,42,43]. Furthermore, it is known that workaholism (in some instances) develops as an attempt to reduce uncomfortable feelings of anxiety and depression. Working hard is praised and honored in modern society, and thus serves as a legitimate behavior for individuals to combat or alleviate negative feelings–and to feel better about themselves and raise their self-esteem [10,11]. Consequently, it is hypothesized that there will be a positive association between anxiety, depression, and workaholism (Hypothesis 4). Against this background, data were analyzed from a large sample in order to investigate whether individual and work-related demographics and psychiatric symptoms in terms of ADHD, OCD, anxiety, and depression could predict workaholism (Hypotheses 1–4).

## Methods

### Procedure

A link to a cross-sectional survey was published in the online editions of five national Norwegian newspapers during the first half of 2014. Participants were informed about the study before providing their answers, and that they were offered instant feedback about their personal workaholism score in return for their participation. Since all data were gathered anonymously and no intervention was performed, signed consent is not demanded according to Norwegian legislation. All responses were saved on a server managed by a specialized survey agency (i.e., www.surveyxact.no). The data were forwarded to the research team after the survey had been promoted for about a week on each newspaper website. Only completed surveys were retained in the final data file. The ethical standards outlined in the Declaration of Helsinki and the Norwegian Health Research Act were followed in the conduct of the study. The Institutional Review Board of the Faculty of Psychology, University of Bergen, Norway, approved the study.

### Sample

The sample comprised a total of 16,426 respondents. Their mean age was 37.3 years (SD = 11.4), ranging from 16 to 75 years. Table 1 provides a detailed overview of the sample.

**Table 1:** Descriptive sample statistics (N = 16,426).

Variable		n	%
**Age**	16–25 years	2898	17.6
	26–35 years	4654	28.9
	36–45 years	4625	28.2
	46–55 years	3082	18.8
	56 years and older	1067	6.5
**Gender**	Female	10487	63.8
	Male	5939	36.2
**Relationship status**	In a relationship	11831	72.0
	Not in a relationship	4595	28.0
**Education**	Primary school	782	4.8
	High school	3413	20.8
	Vocational school	3010	18.3
	University—Bachelor	6045	36.8
	University—Master	2933	17.9
	University—PhD	243	1.5
**Work status**	Full-time worker	12961	78.9
	Part-time worker	3465	21.1
**Position**	Top-level manager	1332	8.1
	Mid-level manager	2714	16.5
	Other managerial tasks	3628	22.1
	No managerial tasks	8752	53.3
**Work sector**	Public	7192	43.8
	Private	8544	52.0
	Self-employed	690	4.2
**Gross income^**a**^**	0–99.999 NOK	778	4.7
	100.000–199.999	941	5.7
	200.000–299.999	1250	7.6
	300.000–399.999	3035	18.5
	400.000–499.999	4336	26.4
	500.000–599.999	2675	16.3
	600.000–699.999	1269	7.7
	700.000–799.999	768	4.7
	800.000–899.000	459	2.8
	900.000–999.999	270	1.6
	1 million or more	645	3.9

^a^Past year personal annual income before tax in Norwegian currency (i.e., NOK).

### Instruments

#### Demographics

Forced choice questions about *age* (year of birth alternatives from 1 = 1997 to 98 = 1900), *gender* (1 = male, 2 = female), *relationship status* (1 = married/common law partner/partner/boyfriend/girlfriend, 2 = single/divorced/separated/widow/widower), *highest completed education* (1 = elementary school, 2 = high school, 3 = vocational school, 4 = Bachelor’s degree, 5 = Master’s degree, 6 = PhD), *professional position* (1 = top-level manager, 2 = mid-level manager, 3 = other manager functions, 4 = no managerial duties), *work sector* (1 = public, 2 = private, 3 = franchise/self-employed), and the past year *gross annual income* (11 alternatives from 1 = 0–99.999 to 11 = 1 million NOK or more) were asked. Participants were also asked to endorse one of several fixed response alternatives regarding *primary occupational status*: 1 = working full-time, 2 = working part-time (followed by an open field for adding specific percentage of full-time equivalent), 3 = retired, 4 = student, 5 = disability pension, 6 = work assessment allowance, 7 = unemployed, 8 = homemaker, 9 = other. Only participants endorsing for working full-time or part-time were included in the present study.

#### Bergen Work Addiction Scale (BWAS)

This scale contains seven items reflecting core addiction elements [13] (i.e., salience, conflict, mood modification, withdrawal, tolerance, problems, and relapse) [10]. Each question is answered on a 5-point scale ranging from *never* (1) to *always* (5), thus yielding a score from 7 to 35, and concerns experiences during the past year (e.g., “How often during the last year have you worked so much that it has negatively influenced your health?” or “…experienced that others have told you to cut down on work without listening to them?”). High scores indicate having workaholism symptoms. Scoring 4 (often) or 5 (always) on four out of seven criteria indicates workaholism. This polythetic cut-off for categorization has been validated in previous research [13–15]. The Cronbach’s alpha for the BWAS was .86 in the present study.

#### Adult ADHD Self-Report Scale (ASRS-Version 1.1)

This scale comprises 18 questions (six main items in part A, and 12 additional items in part B) or symptoms of ADHD in adults [44], based on DSM criteria [9]. All items are answered on a 5-point scale ranging from *never* (1) to *very often* (5), yielding an overall score from 18 to 90 (e.g., “How often do you have trouble wrapping up the final details of a project, once the challenging parts have been done?” or “How often do you have difficulty unwinding and relaxing when you have time to yourself?”). High scores indicate having ADHD symptoms. Scoring 3 (sometimes) or more on item 1–3 and 4 (often) or more on item 4–6 in part A indicates clinical levels of ADHD. Cronbach’s alpha for the ASRS-v1.1 was .86 in the present study.

#### Obsession-Compulsive Inventory-Revised (OCI-R)

This scale comprises 18 items assessing six common OCD symptoms [45]. These include checking (e.g., “I repeatedly check doors, windows, draws, etc.”), ordering (e.g., “I need thing to be arranged in a particular way”), neutralizing (e.g., “I feel compelled to count while I am doing things”), washing (e.g., “I sometimes have to wash or clean myself simply because I feel contaminated”), obsessing (e.g., “I find it difficult to control my own thoughts”), and hoarding (e.g., “I avoid throwing things away because I am afraid I might need them later”). All items are answered on a 5-point scale from *not at all* (0) to *extremely* (4). High scores indicate being bothered by OCD symptoms. Cut-score for clinical levels of OCD is set to 22 or more for the whole scale. The Cronbach’s alphas for the six subscales of the OCI-R in the present study were .72, .79, .65, .62, .84, and .77, respectively (.86 for the whole scale).

#### Hospital Anxiety and Depression Scale (HADS)

This 14-item two-factor scale measures non-vegetative symptoms of anxiety (HADS-Anxiety) and depression (HADS-Depression) [46,47]. Seven items assess levels of anxiety (e.g., “Worrying thoughts go through my mind”), and seven items assess levels of depression (e.g., “I have lost interest in my appearance”). All items are answered along a 4-point frequency scale ranging from *0* to *3*. Cut-score set to 11 or more suggests at least moderate levels of anxiety and depression, and was used in the present study [46]. Cronbach’s alphas for HADS-Anxiety and HADS-Depression in the present study were .81 and .73, respectively.

### Statistics

Firstly, descriptive statistics in terms of internal consistencies, means, and standard deviations were calculated. The prevalence of workaholism was calculated (in accordance with the polythetic criterion), as well as how many workaholics who met the screening cut-off for borderline to clinical levels of OCD, ADHD, anxiety, and depression.

Secondly, Pearson product-moment correlation coefficients were calculated in order to assess the interrelationships between the composite scores of the study scales. In order to better facilitate the practical significance and interpretation of results, both statistical tests for significance (*p* values) and effect sizes (strengths of associations) were evaluated. According to convention, small, medium and large effect sizes correspond to correlation coefficients (r) of .1, .3 and .5, respectively. (This rule of thumb also applies for ß coefficients in regressions [48]).

Following this, a linear multiple hierarchical regression analysis was conducted. Socio-demographic variables and measures of psychiatric symptoms (continuous scores) were regressed upon the composite workaholism score. Basic demographic variables (age, gender, relationship status, and education) were entered in the first step. Work-related variables (work status, position, sector, and gross income) were entered in the second step of the regression analysis. Measures of mental health variables (ADHD, OCD (washing, obsessing, hoarding, ordering, checking, and neutralizing), anxiety, and depression) were entered in the third and final step of the regression analysis. For the variables of education, position, and sector (dummy coded), the largest group comprised the reference category (i.e., Bachelor’s degree, non-manager position, and private sector). According to common effect sizes indices, multiple regression coefficients (R^2^) of about .02, .13 and .26 were benchmarked as small, medium and large effects, respectively (i.e., Cohen’s f^2^ (R^2^/1-R^2^) of .02, .15 and .35) [48].

Additionally, logistic (crude and adjusted) regression analyses were carried out, where workaholism (0 = non-workaholic, 1 = workaholic) comprised the dependent variable, and where socio-demographics and the psychiatric symptoms (ADHD, OCD, anxiety, depression), the latter dichotomized according to the aforementioned cut-offs, comprised the independent variables. In the crude analyses, each of the independent variables was entered separately, exploring the bivariate association between the independent and the dependent variable. In the adjusted analysis, all the independent variables were entered simultaneously, exploring the multivariate associations between the independent variables and the dependent variable. The odds ratio (OR) can be considered as significant when the 95% confidence interval (CI) does not include 1.00.

Preliminary analyses ensured that there was no violation of the assumptions of normality, linearity, multicollinerarity (tolerance over .10 and VIF under 5), and homoscedasticity. The dataset is available as a Data S1 File.

## Results

### Group differences

The prevalence of workaholism in the current sample was estimated to 7.8% (n = 1,287) when using a polythetic approach (i.e., scoring 4 (often) or 5 (always) on at least four of the seven items); and 0.7% (n = 114) when using a monothetic approach (i.e., scoring 4 or 5 on all seven items). The polythetic approach is in line with modern psychiatric nosology. Using the polythetic acoring of the individuals classified as workaholics, the following results were found: 32.7% (n = 421) met the screening cut off for clinical levels of ADHD, whereas 12.7% of non-workaholics met the clinical ADHD-level (χ^2^_df = 1, N = 16426_ = 389.33, *p* < .001); 25.6% (n = 329) met the clinical levels for OCD (8.7% of non-workaholics) (χ^2^_df = 1, N = 16426_ = 369.31, *p* < .001); 33.8% (n = 435) met the clinical levels for anxiety (11.9% of non-workaholics) (χ^2^_df = 1, N = 16426_ = 481.58, *p* < .001); and 8.9% (n = 114) met the clinical levels for depression (2.6% for non-workaholics) (χ^2^_df = 1, N = 16426_ = 150.95, *p* < .001). Hence, there were significant differences between workaholics and non-workaholics on all four clinical states.

### Correlations and descriptive statistics

Table 2 displays mean scores and standard deviations for study variables and their interrelationships. Overall, the internal consistencies for the study scales met satisfactory standards. Two of the OCD subscales (washing and neutralizing) had arguably low Cronbach’s alphas (.62 and .65), but should be noted that these comprised only three items. Furthermore, there were positive and significant correlations between all study scales, ranging from .16 to .59 (i.e., from small to large effect sizes). Workaholism displayed coefficients between .16 (washing) (i.e., small effect size) and .36 (ADHD) (i.e., medium to large effect size), having the strongest association with ADHD, anxiety (.34), obsessing (.26), and depression (.23).

**Table 2:** Descriptive data and correlation coefficients between study variables (N = 16,426).

	Variables	1	2	3	4	5	6	7	8	9	10
**1**	**Workaholism**	—									
**2**	**ADHD**	.36**	—								
**3**	**OCD-Washing**	.16**	.23**	—							
**4**	**OCD-Obsessing**	.26**	.47**	.37**	—						
**5**	**OCD-Hoarding**	.19**	.29**	.22**	.28**	—					
**6**	**OCD-Ordering**	.20**	.24**	.41**	.37**	.26**	—				
**7**	**OCD-Checking**	.20**	.29**	.38**	.41**	.33**	.42**	—			
**8**	**OCD-Neutralizing**	.19**	.26**	.40**	.39**	.25**	.43**	.42**	—		
**9**	**Anxiety**	.34**	.55**	.20**	.59**	.20**	.29**	.34**	.25**	—	
**10**	**Depression**	.23**	.38**	.15**	.44**	.21**	.21**	.22**	.19**	.53**	—
	**M**	14.43	43.31	1.07	1.88	2.37	2.16	2.09	1.76	6.27	3.81
	**SD**	5.50	9.28	1.66	2.40	2.36	2.33	2.21	1.54	3.73	2.99
	**Range**	7–35	18–90	0–12	0–12	0–12	0–12	0–12	0–12	0–21	0–21
	**Alpha**	.86	.86	.62	.84	.77	.79	.72	.65	.81	.73
	**Items**	7	18	3	3	3	3	3	3	7	7

M, mean; SD, standard deviation; ADHD, attention-deficit/hyperactivity disorder; OCD, obsessive-compulsive disorder.

**p < .01.

### Regression analyses

The results from the linear hierarchical regression analysis are presented in Table 3. Individual demographics (age, gender, relationship status, and level of completed education) were entered at Step 1, and only explained 1.2% of the variance in workaholism (F_7,16418_ = 29.62, *p* < .001), with an f^2^ of .01 (i.e., insubstantial effect). Age (ß = -.09), relationship status (ß = .02), high school (ß = -.02), vocational school (ß = -.03), and Master/PhD (ß = .05) contributed significantly to this step. Age and higher education contributed the most.

**Table 3:** Results from the hierarchical regression analysis where individual, work-related, and psychiatric variables were regressed upon the workaholism score (N = 16,426).

	Step 1					Step 2					Step 3				
Variables	(Individual demographics)					(Work demographics)					(Psychiatric symptoms)				
	B	SEB	β	t	p	B	SEB	β	t	P	B	SEB	β	t	p
**Individual demographics**															
Age	-0.043	.004	-.088	-11.014	.000	-0.074	.004	-.153	-17.800	.000	-0.040	.004	-.082	-10.091	.000
Gender (male = 1, female = 2)	0.029	.090	.003	0.319	.749	0.874	.096	.076	9.065	.000	0.892	.090	.078	9.947	.000
In a relationship (yes = 1, no = 2)	0.271	.096	.022	2.820	.005	0.524	.094	.043	5.569	.000	.463	.086	.038	5.403	.000
Primary school^a^	-0.048	.209	-.002	-0.228	.820	0.380	.208	.015	1.824	.068	-0.193	.189	-.007	-1.022	.307
High school^a^	-0.283	.119	-.021	-2.385	.017	0.013	.120	.001	0.111	.911	-0.171	.109	-.013	-1.577	.115
Vocational school^a^	-0.444	.124	-.031	-3.589	.000	-0.218	.122	-.015	-1.780	.075	-0.325	.111	-.023	-2.929	.003
University Master/PhD^a^	0.697	.120	.050	5.809	.000	0.406	.120	.029	3.378	.001	0.357	.109	.026	3.272	.001
**Work demographics**															
Work status (full-time = 1, else = 0)						-0.007	.117	-.001	-0.062	.950	0.134	.106	.010	1.259	.208
Top-level manager position^b^						3.128	.175	.155	17.875	.000	3.179	.159	.158	20.050	.000
Mid-level manager position^b^						2.158	.123	.146	17.571	.000	2.265	.111	.153	20.353	.000
Other managerial tasks^b^						1.183	.107	.089	11.033	.000	1.222	.097	.092	12.589	.000
Public sector^c^						-0.323	.092	-.029	-3.501	.000	-0.125	.084	-.011	-1.494	.135
Franchise/self-employment^c^						1.017	.218	.037	4.666	.000	0.791	.197	.029	4.012	.000
Annual gross income						0.222	.027	.089	8.088	.000	0.282	.025	.113	11.319	.000
**Psychiatric symptoms**															
ADHD											0.116	.005	.196	22.745	.000
OCD-Washing											0.116	.027	.035	4.307	.000
OCD-Obsessing											-0.019	.022	-.008	-0.880	.379
OCD-Hoarding											0.152	.018	.065	8.454	.000
OCD-Ordering											0.123	.020	.052	6.257	.000
OCD-Checking											0.030	.021	.012	1.450	.147
OCD-Neutralizing											0.145	.030	.041	4.901	.000
Anxiety											0.243	.015	.165	16.527	.000
Depression											0.106	.016	.058	6.810	.000
**Model summary**															
Variance explained by model	R^2^ = .012 (1.2%)					R^2^ = .066 (6.6%)					R^2^ = .237 (23.7%)				
Change in variance by next step						ΔR^2^ = .054 (5.4%)					ΔR^2^ = .170 (17.0%)				
Statistical significance of model	F (7, 16418) = 29.618, p = .000					F (14, 16411) = 83.448, p = .000					F (23, 16402) = 221.250, p = .000				
Statistical significance of steps						ΔF (7, 16411) = 135.578, p = .000					ΔF (9, 16402) = 406.727, p = .000				

B, unstandardized regression coefficient; SEB, unstandardized standard error; β, standardized regression coefficient; t, t-test value; p, probability value

R^2^, squared multiple correlation coefficient; ΔR^2^, change in R^2^ between steps; F, F value with corresponding degrees of freedom; ΔF, change in F between steps.

^a^Bachelor’s degree comprises the reference category.

^b^Non-managerial position comprises the reference category.

^c^Private sector comprises the reference category.

Work demographics (work status, position, sector, and annual gross income) entered at Step 2, additionally explained 5.4% of the variance, ΔR^2^ = .054, ΔF_7,16411_ = 135.58, *p* < .001, with an f^2^ of .06 (i.e., small effect). Top- and mid-level manager positions contributed the most among the work variables. After controlling for work demographics, age (ß = -.15), gender (ß = .08), relationship status (ß = .04), and Master/PhD (ß = .03) were significant, whereas high school and vocational school became non-significant. Of the work variables, top-level management (ß = .16), mid-level management (ß = .15), other managerial duties (ß = .09), public sector (ß = -.03), franchise/self-employment (ß = .04), and income (ß = .09) contributed significantly to this step. The overall effect of individual and work demographics on workaholism was still relatively small (f^2^ = .07).

Psychiatric symptoms (ADHD, OCD symptoms, anxiety, and depression) entered at Step 3 explained 17.0% of the variance, ΔR^2^ = .170, ΔF_9,16402_ = 406.73, *p* < .001. ADHD and anxiety contributed the most, and the psychiatric variables had a much more substantial effect on workaholism (f^2^ = .21; medium effect size). Following entry of all independent variables at Step 3, the total variance explained by the model as a whole was 23.7%, F_23,16402_ = 221.25, *p* < .001, with an f^2^ of .31, which is considered as a large effect. In the final model, age (ß = -.08) and vocational school (ß = -.02) were negatively associated with workaholism, while gender (being female, ß = .08), relationship status (being single, ß = .04), having a Master’s/PhD (ß = .03), top-level management (ß = .16), mid-level management (ß = .15), other managerial duties (ß = .09), franchise/self-employment (ß = .03), high income (ß = .11), ADHD (ß = .20), washing (ß = .04), hoarding (ß = .07), ordering (ß = .05), neutralizing (ß = .04), anxiety (ß = .17), and depression (ß = .06), were all positively associated with scores on workaholism. Overall, the effect sizes for each variable were relatively small–except for ADHD and anxiety, displaying coefficients (ß) that may be considered as medium-sized effects.

Table 4 presents the results from the logistic regression analyses in terms of odds ratio (OR) and 95% confidence intervals (95% CI) for both the crude and the adjusted analyses. For dummy coded variables (i.e., education (Bachelor’s degree), position (non-manager), and sector (private)), the largest group comprised the reference for which the OR is set to 1.00. In both the crude and adjusted analyses, workaholism cases were inversely related to age, but positively related to being female, single status, manager positions, self-employment, and clinical levels on all four psychiatric variables. Income was unrelated to workaholism in the crude analysis, but became positively and significant in the adjusted analysis. On the other hand, workaholism was inversely related to public sector in the crude analysis, but did not remain significant when controlling for the other variables in the adjusted analysis. Of note, workaholism was unrelated to education and work status (full-time vs. part-time employment) in both analyses.

**Table 4:** Logistic regression analysis where workaholism (0 = non-workaholic, 1 = workaholic) comprised the dependent variable, and where socio-demographics and cut-off based psychiatric symptoms comprised the independent variables (N = 16,426).

	Crude analysis			Adjusted analysis		
Variable	OR	95% CI	p	OR	95% CI	p
**Age**	0.971	0.966–0.976	.000	0.965	0.959–0.972	.000
**Gender** (1 = male, 2 = female)	1.212	1.073–1.368	.002	1.765	1.528–2.038	.000
**In a relationship** (1 = yes, 2 = no)	1.342	1.189–1.515	.000	1.354	1.189–1.542	.000
**Bachelor degree** (reference group)	1.00			1.00		
**Primary school**	1.250	0.965–1.620	.091	1.002	0.753–1.334	.989
**High School**	1.115	0.956–1.300	.166	1.002	0.845–1.189	.978
**Vocational school**	0.892	0.752–1.058	.189	0.961	0.799–1.154	.667
**University Master/PhD**	1.087	0.928–1.274	.301	1.015	0.856–1.204	.865
**Work full-time** (1 = yes, 0 = no)	1.083	0.940–1.249	.270	1.042	0.876–1.238	.645
**Non-manager** (reference group)	1.00			1.00		
**Top-level manager**	2.339	1.949–2.807	.000	2.997	2.396–3.749	.000
**Mid-lever manager**	1.933	1.665–2.244	.000	2.447	2.072–2.891	.000
**Other managerial tasks**	1.367	1.178–1.586	.000	1.575	1.345–1.846	.000
**Private sector** (reference group)	1.00			1.00		
**Public sector**	0.781	0.692–0.881	.000	0.936	0.817–1.071	.336
**Franchise/self-employed**	1.751	1.392–2.203	.000	1.367	1.059–1.763	.016
**Annual gross income**	1.023	0.997–1.049	.081	1.132	1.091–1.175	.000
**ADHD** (1 = not, 2 = case)	3.355	2.958–3.805	.000	2.260	1.963–2.601	.000
**OCD** (1 = not, 2 = case)	3.586	3.126–4.114	.000	2.205	1.883–2.583	.000
**Anxiety** (1 = not, 2 = case)	3.779	3.333–4.284	.000	2.422	2.091–2.805	.000
**Depression** (1 = not, 2 = case)	3.609	2.906–4.482	.000	1.555	1.213–1.993	.000

OR, odds ration; CI, confidence interval; p, probability value.

The full model containing all predictors (adjusted analysis) was statistically significant (χ^2^_df = 18, N = 16426_ = 1029.08, *p* < .001). Furthermore, the model as a whole explained between 6.1% (Cox and Snell R square) and 14.4% (Nagelkerke R Squared) of the variance in workaholism status and correctly classified 92.2% of all cases. In the final model the proportion of corrected classified cases did not increase from the null model.

## Discussion

The present large-scale study substantially extends the literature on work addiction by investigating the associations between workaholism and different symptoms of psychiatric disorders. The results, involving self-ratings from more than 16,400 adults, were derived from one of the largest surveys ever undertaken in the area (based on number of participants). Taken together, the symptoms of ADHD, OCD, anxiety, and depression contributed significantly to the variance in workaholism (17%)–after controlling for socio-demographics, which alone explained 6.6% of the variance in the linear hierarchical multiple regression model. The prevalence of workaholism in this sample was estimated to be 7.8%, and is in accordance with recent estimation (8.3%) in a nationally representative sample of Norwegian employees [14], and appears to be similar to the 10% estimate presented in a comprehensive review [18]. Workaholics scored significantly higher on clinical levels of all psychiatric symptoms than non-workaholics. Although the following discussion will focus on the results from the final step of the hierarchical regression, and the results regarding the categorical clinical independent variables in the adjusted logistic regression analysis, the section begins with a short evaluation of findings concerning workaholism and socio-demographic variables.

### Individual and work-related demographics and workaholism

Workaholism was associated with specific characteristics. In general, younger, being single, highly educated people with higher socioeconomic status tended to report higher levels of workaholism than their comparison groups. Workaholism was also more prevalent among managers, self-employed, and people working in private sector, compared to non-managers, and public sector. These results were for the most part expected (Hypothesis 1), and concur with results from the few previous studies examining similar variables [2,13,14,19,20,22,23,39]. Although education contributed positively in the linear regression, especially before entering work and psychiatric variables, it did not influence workaholism cases in the logistic regression analyses. No effect of gender was found in the initial step of the linear regression analysis, again confirming the findings of previous research [2,14,19]. However, in both the final step of the linear regression analysis and in the adjusted logistic regression analysis being female was significantly associated with workaholism. This might reflect that women might have higher ambitions than men and the result is in line with studies showing that (in recent years) women now outperform men regarding grades in higher education in Norway [49]. Although previous research has primarily reported workaholism to be unrelated to relationship status [20], in the present study an association was found in both the linear and the logistic regression analysis, that individuals not in a relationship were more likely to be workaholics. One possible explanation may be that workaholics avoid or break off relationships, or their partners leave them, due to their excessive working.

Overall, the results of the present study indicate that specific socio-demographic groups may be at risk of developing workaholism. However, the results may also reflect that workaholics choose positions, jobs, and/or sectors that allow them to engage in their preferred day-to-day working practices. The growing body of research tends to show that workaholism affects younger (rather than older) adults. This might be interpreted as a cohort-effects suggesting that workaholism is on the rise. Alternatively, it may reflect an age-effect suggesting that problems with workaholism decrease as the person becomes more mature [14]. However, it might simply reflect adjustments people make or are forced to make (e.g., poorer health) and obligations that come with age (e.g., having a family). This is in line with a recent longitudinal study showing that workaholism decreased during a person’s career [50], as well as the current findings indicating that people in a relationship are slightly less likely to experience workaholism than those who are single. The presence or absence of children within a relationship may also be a factor. However, a recent study reported that workaholism did not differ according to the number of children [51]. Of note, the explained variance by individual (1.2%) and work demographics (5.4%) was very small in the linear regression, thus having no practical implications, beyond age and leadership positions.

### Attention-Deficit/Hyperactivity Disorder and workaholism

Symptoms of ADHD were positively related to workaholism, and had a slightly greater effect on workaholism than the other psychiatric symptoms in both the correlation and linear regression analyses. The logistic regressions also showed high odds ratios for workaholism among those categorized with clinical levels of ADHD. Among workaholics, 32.7% met the screening cut-off for clinical ADHD levels. These findings are in line with established knowledge of the co-occurrence of ADHD and addictions in general [26], although the present study is the first ever to associate work addiction with ADHD, thus providing support for the second hypothesis. Although ADHD is often associated with unemployment and being unable to conduct normal work [26], the present authors’ hypothesized that ADHD would be related to workaholism partly for this very same reason. Individuals with ADHD may have to work harder and longer to compensate for their work behavior caused by neurological deficits. They may also be at risk of taking on projects and tasks impulsively–resulting in more work than they can realistically do within normal working hours. Some, but far from all, with this disorder are also very hyperactive [8,26]. Hence they may choose and thrive better in jobs with frequent deadlines and higher levels of work stress, conditions that may alleviate their inner restlessness (e.g., self-medication).

The present authors also propose that such people are unable to relax, and may keep on working nonstop–if they find a task interesting and demanding enough (e.g., hyper-focus). Furthermore, it is hypothesized that these workaholic ADHD types push themselves in their job in order to disprove conceptions of them by others as being lazy or unintelligent. History portrays many highly successful entertainers, inventors and entrepreneurs, authors and scientists as well as business leaders with ADHD traits–often associated with hard working talent and abundant creativity [52]. Given that the first academic writings on workaholism appeared in the early 1970s [53], it is arguably surprising that the present study is the very first that empirically link symptoms of ADHD with workaholism. This may be because ADHD is often thought of as a child disorder from which sufferers grow out of before reaching adulthood [26]. This is now known not to be the case, and ADHD is probably under-diagnosed in adults [26]. Instead, such adult individuals are often diagnosed with bipolar disorder, anxiety, depression, borderline personality disorder, etc. [26]. The current findings are also in accordance with several popular workaholic typologies portrayed in recent years [31].

### Obsessive-Compulsive Disorder and workaholism

All OCD symptoms were positively related to workaholism in the correlation analysis. However, after controlling for all other study variables, the associations changed both in magnitude and direction in the linear regression analysis. Washing, hoarding, ordering, and neutralizing, were still positively related to workaholism, whereas obsessing and checking became non-significant. Together, the association between OCD symptoms on workaholism was relatively weak overall. Among workaholics, 25.6% met the screening cut off for clinical levels of OCD and in the adjusted logistic regression analysis those scoring above the OCD-cutoff had a significant and elevated risk of being categorized as workaholics. Taken together, the study’s third hypothesis received some support although some findings regarding particular OCD-symptoms were not in line with the hypothesis.

Why obsessing did not contribute positively to the linear regression model is difficult to explain, as many scholars believe obsession is a critical component in defining workaholism [54] and obsession can play a major role in the developing of addictions more generally [10]. Obsessive checking was also expected to (at least in part) explain workaholism, as controlling and checking have been regarded by some scholars as central aspects of workaholism [55]. However, this also turned out non-significant in the linear regression analysis. One explanation for the unexpected findings regarding obsessing and checking may be related to the other variables included in the analyses which may have caused inhibition effects due to the positive correlation between all the independent variables. Despite these partly unexpected findings, OCD symptoms were generally positively related to workaholism (i.e., four out of six subscales) in the linear regression as well as significantly related to workaholism in the logistic regression analysis. These findings are also in line with previous workaholic typologies [31,37] and previously empirically demonstrated relationships [2,30,40].

### Anxiety and depression and workaholism

Both anxiety and depression were positively related to workaholism in the correlation analysis, as well in the both regression analyses–lending support to the fourth hypothesis and are in keeping with previous research [2,42,43]. However, anxiety only had a small effect on workaholism in the linear regression analysis, whereas depression demonstrated a trivial effect. This may suggest that there are few significant practical implications. Nevertheless, scoring above clinical cut-offs for anxiety and depression was clearly related to workaholism in the logistic regression analysis. These findings are in agreement with the fact that addictive behaviors, depression, and anxiety often co-occur [33,36].

The results perhaps suggest that workaholics are more anxious than depressed. Among workaholics, 33.8% met the screening cut-off for borderline or clinical anxiety level, whereas the corresponding percentage for clinical levels of depression was 8.9%. Workaholism has also previously been linked to the personality trait of neuroticism (e.g., being anxious, fearful, moody) [14,22]. People with high scores on this trait often handle stressors poorly [56]. Consequently, quite ordinary work tasks and working situations may be perceived as threatening and overwhelming, thus motivating anxious individuals to cope with these threats by allocating extra time and effort to task completion. Therefore, the present authors propose that working may act as an escape mechanism related to feelings of anxiety and depression [10,36]. Another explanation for the findings could also be that anxious people fear failing (and go over their work several times) and/or decline incoming tasks (overload), whereas depressed people work slower (due to low energy level) and have to compensate by working longer hours to get the work done.

### Practical implications

In the present study, several potential risk factors for workaholism were identified that suggest some practical implications. Firstly, organizational interventions should aim to prevent and help young adults and managers in how to suppress and inhibit workaholic tendencies and maintain a positive ‘work-life’ balance. This is particularly important in areas with an excessive work climate, as studies have shown that both personal and organizational characteristics–as well as cultural characteristics–are involved when workaholics are “made” [23,39,40,57].

Although workaholism has become an increasingly studied area for empirical investigation over the last decade, clinical interventions are still few and far between [6]. However, interventions using approaches in line with validated therapies for addictions in general may be feasible [58]. Such approaches typically involve a collection of self-help techniques, psychotherapies, and pharmacological assistance [58]. Relevant interventions may thus involve cognitive-behavioral therapy (CBT) and motivational interviewing (MI) techniques–the two most commonly utilized counseling approaches for a broad spectrum of addictions. CBT aims to single out thoughts and emotions that trigger and activate workaholic behavior, and replace these with a more sufficient mindset [59]. Training in stress and time management to better cope with distress and fight-and-flight states may also be helpful (e.g., relaxation techniques, mindfulness meditation) [32,60,61]. MI aims to awakening inner motivation to pursue positive behavioral changes from the workaholic. For this purpose, the therapist typically uses a set of communication techniques to uncover and dissolve ambivalence. The method seeks to engage the workaholic, bring out change talk, and induce motivation to change current behavior [62].

Secondly, the present study highlights the relevance of further investigating underlying neurobiological deviations related to the workaholic behavior–as significantly more workaholics met the clinical levels of ADHD, OCD, anxiety, and depression than their non-workaholics counterparts. If there are biological bases to the addiction, medication might be an option. Some research has indicated that specific medications (e.g., Bupropion, Escitalopram, Methylphenidate) are useful in treating other behavioral addictions [63–66].

As very little empirical focus has been on adult ADHD, practitioners have often relied on diagnostic criteria for children found in the DSM-IV [9,26]. One criterion is that the symptoms must cause some impairment in two or more settings (e.g., home and school). Another is that there must be clear evidence of clinically significant impairment in social, academic, and/or occupational functioning [8]. Still, it is unlikely that many workaholics are being screened for ADHD [26]. However, it is not unthinkable that for workaholics with ADD (inattentive type), work is the only area of their lives they master–with significant impairment of all other life domains (e.g., household, relationships, health and well-being) (e.g., criteria of two or more impaired domains). Handling a job is crucial in most cultures–therefore, this type of ADD affected worker may struggle to fulfill this highly visible sociocultural function, spending much time and energy to accomplish work (that is expected) that others get done within normal working hours. In line with this, previous research has shown that workaholism is associated with impaired job performance [4,5]. Furthermore, a workaholic with ADHD (hyperactive/impulsive type) might end up in jobs involving extreme pressure, such as a foreign journalist in a war zone. Sadly, due to overlooked ADHD symptoms by physicians, some of these individuals may be missing out of living a full life–as they often are left with work-life conflicts, troubling social lives, chaotic domestic lives, and poorer performance than they with professional help might otherwise have had [26]. Fortunately, DSM-5 [8] combats this by refining ADHD criteria to include adult symptoms.

Clearly, more research is warranted to elucidate these important relationships further. In the meantime, it is recommended that physicians and therapists should not take for granted that a seemingly successful workaholic do not have ADHD-related clinical features. However, more research is needed to examine whether workaholism is totally negative for all individuals as it may be that workaholism may serve an important structuring function for those with mental health problems and those with social dysfunction.

### Strengths and limitations

The present study is not without some limitations. Due to the very large sample size and statistical power to the analyses, some trivial relationships may have turned out significant. For example, at Step 1 in the linear regression analyses, only 1.2% of the variance was explained. To better enable researchers to draw conclusions regarding whether or not the effects are nontrivial in size, effect sizes were also calculated and reported. Thus, instead of only reporting the statistical significance, the focus on effect sizes may facilitate and communicate the more practical significance of the study’s findings. Another limitation concerns the cross-sectional design, as directionality and causality cannot be established. It is also possible that workaholism may predict inattention, anxiety and depressive symptoms. Hence, the relationships between anxiety, depression and workaholism, in particular, may well be the reverse than that portrayed in the present study [36]. The directionality between study variables would be revealed by the use of longitudinal study designs in the future although such designs are often very resource demanding.

Additionally, as all data collected in the present study relied on self-report, common method biases may have affected the results [67] including recall biases and social desirability biases. Another limitation of the present study involved the open web-based convenient sampling methodology. Consequently, nothing is known about non-responders of this self-selecting sample, and the survey might have attracted or repelled specific groups or individuals. For example, there was a higher proportion of women in the present sample, and therefore self-selection might have influenced the findings.

Overall, this puts limitations on the generalizability of the findings to other populations both within and outside of Norway–making it inapplicable to estimate population characteristics. Regardless of such limitations, the present study is considered suitable for estimations of relationships between characteristics and variables under investigation [68]. Another potential limitation concerns the fact that some participants might not have taken their participation seriously and provided random answers. However, the reliability indexes suggest that the scales overall appeared to have been completed consistently.

In terms of strengths, the present study had a large sample size, providing a high level of statistical power to the analyses. Although ADHD has previously been linked to several other addictions [26] the present study is the very first that empirically links ADHD with workaholism. The combination of variables included in the present study is also new to the field. Also, all the scales used in the present study were internally consistent at large, previously validated, and psychometrically robust. Furthermore, they were embedded within contemporary addiction theories [10,13,14,44, 45,47]. Finally, it should be noted that the newspapers that published the survey represent very different reader groups, and they are nationwide instead of more localized ones.

## Conclusions

The present study suggests that having symptoms of an underlying psychiatric disorder is associated with workaholism. A synthesis of individual (1.2%), work-related (5.4%), and mental health (17.0%) variables, explained 23.7% of the variance in workaholism, which is considered a large effect. ADHD, anxiety, lower age, and managerial positions stood out as most consistent and conceptually meaningful in the linear regression. Although gender, relationship status, education, work sector, income, obsessive-compulsive symptoms, and depression also contributed significantly, their effects were weaker and more inconsistent across the steps in the regression model.

According to recommended cut-offs, 7.8% of the present sample was classified as workaholics. Following this, it became evident that individuals that were younger, female, not in a relationship, managers, self-employed, and met clinical cut-offs for ADHD, OCD, anxiety, and depression, were more often categorized as workaholics than their comparison groups. Workers with some of these characteristics could thus be targets for interventions with the aim of preventing the development and maintenance of workaholism. More research preferably using representative and clinical samples, on this poorly studied relationship between workaholism and psychiatric disorder symptoms is clearly needed.

## Supporting Information

S1 File The file contains all relevant data underlying the findings described in this study. (SAV)
